# A Meta-Analysis of Soccer Injuries on Artificial Turf and Natural Grass

**DOI:** 10.1155/2013/380523

**Published:** 2013-06-19

**Authors:** Jay H. Williams, Emmanuel Akogyrem, Jeremy R. Williams

**Affiliations:** Department of Human Nutrition, Foods and Exercise, Virginia Polytechnic Institute and State University, Blacksburg, VA 24060, USA

## Abstract

The goal of this investigation was to determine if playing or training on third-generation artificial turf (AT) surfaces increases the incidence rate of injuries compared to natural grass (NG) surfaces. This was accomplished by a meta-analysis performed on previously published research. Eight studies met the criteria of competitive soccer players, participation on both surfaces, and presentation of both exposure time and injury occurrence. Exposure time and injury incidence values were used to generate injury rate ratios (IRRs, AT/NG) for all injuries as well as specific injuries. Subgroup analyses were also performed by condition (match or training), gender, and age (youth or adult). The overall IRR was 0.86 (*P* < 0.05) suggesting a lower injury risk on AT than NG. However, there was considerable heterogeneity between studies. Analyses of individual injuries and subgroups found that in many cases IRR values were significantly less than 1.0. In no case was the IRR significantly greater than 1.0. Based on this, it appears that the risk of sustaining an injury on AT under some conditions might be lowered compared to NG. However, until more is known about how issues such as altered playing styles affect injury incidence, it is difficult to make firm conclusions regarding the influence of AT on player safety.

## 1. Introduction

Unfortunately, acute injuries are far too common in the sport of soccer. Sprains and ruptures of the ligaments supporting the ankle and knee joints as well as muscle strains occur quite often. In some cases, joint injuries are sustained through contact with another player. A classic example occurs when one player collides with another, applying excessive force to the lateral side of the opponent's knee. This often results in damage to the medial collateral ligament, lateral meniscus, and the anterior cruciate ligament (ACL). However, a substantial number of soccer injuries occur through noncontact mechanisms. In these cases, an athlete may plant his or her foot then stop, cut, or turn. As the body changes direction while the foot is stationary, the knee and/or ankle experiences torque. As a result, ligament structures can be strained or ruptured.

Researchers have identified a number of risk factors for noncontact injuries. These include intrinsic factors such as proprioception, muscular strength, ligament properties, and biomechanics as well as extrinsic factors such as the playing surface and other environmental conditions. Several studies have focused on this latter factor as important, specifically the use of artificial turf playing surfaces. They suggest that the added friction between the shoe and the surface increases the torque experienced by the ankle and knee [1, 2]. This, in turn, raises the risk of a ligament injury. In fact, early studies on the first-generation artificial turf (AT) fields (“Astroturf-type” surfaces) did show an increased injury risk compared to natural grass (NG) [3, 4]. However, the nature of AT has changed over the past 10 years. The short-pile carpet laid over a thin pad has been replaced by a surface that contains long “grass-like” fibers that are embedded with granules of crushed rubber, sand, and/or silica and laid over a thick pad. This results in a more compliant surface and one that results in a considerably lower shoe-surface coefficient of friction [1]. Manufacturers of third-generation AT surfaces argue that such a design may lower the risk of noncontact joint injury. On the other hand, many coaches, trainers, and athletes remain concerned that playing on AT increases injury risk.

Whether or not third-generation AT alters the risk of noncontact injury remains unresolved. Several recent studies have focused on comparing injury incidence rates in soccer players who train and/or play matches on both AT and NG. Unfortunately, the results are somewhat variable, with some studies showing reduced risk of some injuries on AT and others showing no difference or slightly increased risk. Thus, the purpose of this investigation was to resolve this issue. To accomplish this, we used previous research and a meta-analytic approach to compare injury rates in soccer players performing on AT and NG.

## 2. Methods

PubMed and Google Scholar searches were used to identify individual studies. Key search terms were soccer, injury, grass, synthetic turf, and artificial turf. To be included in the meta-analysis, studies met several criteria: (1) focused on competitive soccer players, (2) compared acute injuries sustained during soccer matches and/or training on NG and on third-generation AT surfaces, (3) examined players who participated on both surfaces during the study period rather than teams that used one surface exclusively, (4) used the injury definition as described by Fuller et al. [5], (5) reported both exposure times and injury occurrences for play on AT and NG, and (6) written in English or an English translation.

The studies meeting our criteria were examined and exposure times, injury occurrences, and playing surface were recorded. In addition, the playing condition (match or training), gender, and age (youth or adult) were noted. Values for all acute injuries (regardless of type) were recorded. Chronic injuries were not considered as these could be attributed to both surfaces or to other causes. Acute knee injuries, ankle injuries, foot injuries, sprains, and muscle strains were recorded. These specific locations and types of injuries were selected because they were the most consistently defined across all of the included studies.

Crude injury rates were computed using the unweighted sum of total injuries and exposure times from all eight studies. Statistical analyses were performed on adjusted injury incidence rate ratios (IRRs, AT/NG) using the Mantel-Haenszel method for fixed effects. Upper and lower 95% confidence intervals were also computed. Significance was established at the 0.05 level. IRR values were first computed for entire data set. The data were then categorized based on condition (match or training), gender, and age (youth or adult).

## 3. Results

An initial literature search on PubMed returned more than 25 citations. Of those, eight studies met our criteria. These are shown in Table 1 along with key characteristics of each study. As can be seen, individual studies varied in terms of the number of players and teams examined. In addition, the period of investigation ranged from several months to several years resulting in varying exposure times. Three studies focused on either match or training injuries exclusively while five included both conditions. One study examined females exclusively and one did not categorize their data by gender. Five of the studies focused on adult players, generally college or professional players, whereas three studies focused on youth players that were 12 to 17 years of age.

The eight studies resulted in a total exposure time of 1,498,343 hours. Of that, 571,196 hours were played on AT (34.5%) and 981,147 on NG (65.5%). The amount of time spent in training and playing on AT and NG varied between studies. In the study by Soligard et al. [12], players competed on AT for less than 10% of the total time played. At the other end of the range, Ekstrand et al. [8] studied players who trained and competed on AT nearly three times as long as on NG. Matches accounted for 271,022 hours (18.1%) of total exposure time whereas training accounted for 1,227,321 hours (81.9%). Of the total exposure time, males accounted for 949,568 hours (66.1%) and females for 486,178 hours (33.9%). Soligard et al. [12] did not distinguish between male and female players and was not included in the analysis of gender.

A total of 9737 injuries were recorded with 2670 occurring on AT (27.4%) and 7067 on NG (75.6%). This resulted in crude injury incidence rates of 5.16 and 7.20 injuries per 1000 hours for AT and NG, respectively. Crude incidence rates for matches were 20.26 and 24.45 injuries per 1000 hours for AT and NG. For training injures on AT and NG, the incidence rates were 2.91 and 2.68 injuries per 1000 hours. 

**Figure 1:**
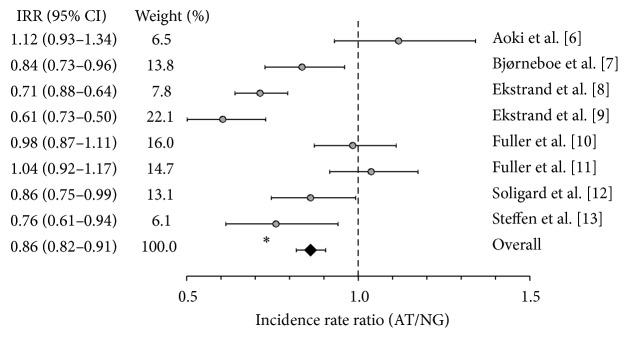
Injury incidence rate ratios for all injuries occurring on AT and NG (95% CI) ∗*P* < 0.05.

Figure 1 shows the injury IRRs for each of the studies examined along with the overall adjusted IRR of 0.86 (0.74–0.93, *P* < 0.05). This analysis included all reported injures and all categories examined. Five of the eight studies showed IRRs that were significantly lower than 1.0, indicating a lower incidence rate on AT [7–9, 12, 13], while the other three showed nonsignificant differences. The overall adjusted IRR was statistically significant with a lower incidence rate on AT.

**Figure 2:**
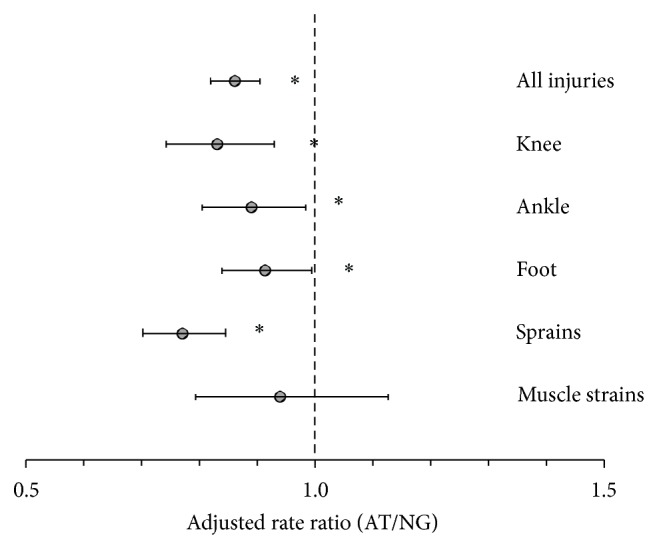
Incidence rate ratios for various injuries occurring on AT and NG (95% CI) ∗*P* < 0.05.

Figure 2 shows the overall IRRs of specific injuries and locations. For knee, ankle, and foot injuries as well as sprains, incidence rates were significantly lower on AT compared to NG. The overall IRR for muscle strains was not significant.

The adjusted IRRs for the subcategory analyses are shown in Table 2. Analyses of male, female, youth, and adult subcategories for all injuries showed IRR values significantly lower than 1.0. For knee injuries, incidence rates on AT were lower for training injuries and adults. For sprains, incidence rates were lower on AT for females and adults. For muscle strains, incidence rates were lower on AT for match injuries, males, and both young and adult players. In no case was injury incidence increased on AT.

**Figure 3:**
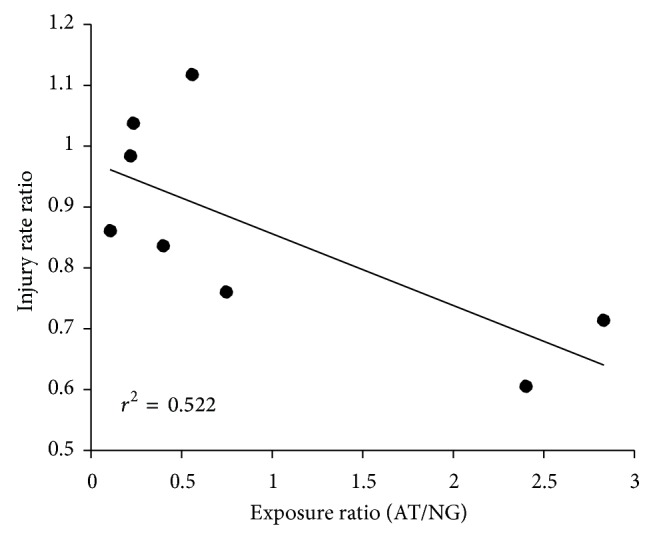
The correlation between the exposure ratio (exposure on AT/NG) and the injury incidence rate ratio. Each data point represents a single study.

There was considerable heterogeneity across the studies examined. For the entire data set, including all injuries as well as both conditions, gender and age (Figure 1), the *I* ^2^ value was 80%. A portion of the heterogeneity was due to different injuries and locations. For specific injuries and sites (Figure 2), *I* ^2^ ranged between 43% and 74%. Condition, gender, and age also contributed as *I* ^2^ values for these categories ranged from 0 to 80%. We found that variations in exposure to AT accounted for some of the heterogeneity.  Figure 3 shows a significant negative correlation between the relative amount of time spent on AT (AT exposure/NG exposure) and the IRR for each study. As can be seen, the two studies in which the greater exposure time on AT had the lowest IRR.

## 4. Discussion

This meta-analysis examined eight studies that compared soccer injury rates occurring on AT and NG. In total, these studies report nearly 1.5 million hours of training and match play and almost 10,000 injuries. The adjusted IRR for all injures was significantly less than 1.0 indicating lower incidence rates for playing and training on AT. For specific categories and specific injuries, several IRR values were less than 1.0. In no case did we find an IRR value significantly greater than 1.0. Thus, the overall results of our analyses do not support the idea that playing or training on AT increases the risk of injury compared to NG. In fact, our analyses suggest that AT surfaces may reduce injury incidence for some types of injuries within specific categorizations. 

There are several criticisms of our study that should be pointed out. First, despite the guidelines provided by Fuller et al. [5], the eight studies varied in terms of their injury descriptions. For example, Ekstrand et al. [8, 9] reported the incidence of injuries based on location and type (e.g., “knee sprains”), whereas Bjørneboe et al. [7], Fuller et al. [10, 11], Soligard et al. [12], and Steffen et al. [13] described injuries by location (e.g., “knee”) or type (e.g., “sprain”). Only Ekstrand et al. [9] noted specific injuries such as “anterior cruciate ligament tears.” Thus, our results are limited to the general classifications of location (knee, ankle, and foot) or type (sprains and muscle strains). Second, in one study [12], exposure and incidence values for males and females were pooled. This study was excluded from the subcategory analysis of gender. Third, the severity of injuries is categorized based on the number of training and match days missed. Unfortunately, there is inconsistency in this method between studies. For example, a “minor” or “mild” injury was defined as 1–7 days [7, 10, 11, 13], 4–7 days [8, 12], and 1–2 weeks [6] missed. Classification of severe injuries also varied from >21 to >28 days missed. Given these discrepancies, we were not confident using severity as a subcategory for analysis. Fourth, the studies failed to report environmental conditions such as temperature, wet/dry field conditions, or shoe type/cleat design. Finally, only three of the studies differentiated between contact and noncontact injuries [10, 11, 13]. This is an important limitation since contact injuries may or may not be directly attributable to playing surface.

Our results do not provide an explanation for possible reductions in injury risk on AT. Artificial turf surfaces provide a more consistent playing surface than NG, free of bare sports, ruts, and divots which could affect injury risk. However, the studies examined focused on teams competing at a high level, so it is likely that the NG surfaces were of high quality. Traditionally, physical characteristics of AT and frictional resistance at the shoe-surface interface have given rise to suggestions of greater injury risk on AT. However, laboratory experiments yield conflicting results regarding this idea. Several studies show greater rotational torque between the shoe and AT compared to NG [2, 14], whereas Cawley et al. [1] show higher values on NG. Livesay et al. [2] further argue that stiffness (the rate of change in torque) may play a role in injury risk. Yet, greater peak torque on AT does not result in increased stiffness. In human studies, Ford et al. [15] found different foot loading patterns during cutting maneuvers performed on AT and NG. On the other hand, Potthast [16] reported no differences in rear foot and ankle movements of the plant foot when goal kicking between NG and rubber-filled AT. McGhie and Ettema [17] found that impact forces experienced during cutting maneuvers on AT were not indicative of a hazardous condition. To further complicate this issue, torque and stiffness can be affected by the type of cleat worn on a particular surface as well as the temperature of the surface [2, 14]. Thus, it is difficult to conclude with confidence which of the physical characteristics of AT accounts for difference in injury incidence rates found in the present study.

It is possible that other factors may impact injury potential. For example, players seem to alter their style of play on AT. Match analyses show that the amount of time spent running at different intensities is similar between surfaces, but fewer slide tackles and shorter passes are executed on AT [18]. The reduction in slide tackles may stem from the fear of skin abrasions but may limit the number of “high risk” situations for injury, particularly contact injuries. Players also have more negative perceptions of playing on AT. They feel that heat as well as speed of play on AT requires greater physical effort compared to grass [18, 19]. However, these perceptions have been difficult to confirm as some controlled studies show increased energy expenditure while others show no differences in fatigue [20–23]. Ronkainen et al. [19] note that negative player perceptions of AT are stronger in those who have limited exposure to AT or are exposed to AT later in their careers.  Figure 3 suggests that more time spent in training and competing on AT is associated with lower injury risk. Perhaps with increased exposure to AT, players develop a less aggressive play with fewer slide tackles that reduces injury risk. It should be pointed out, however, that this idea is speculative at this time. Clearly more research is needed regarding player movements, energy expenditure, and fatigue on AT and NG surfaces.

This study highlights several areas that need to be addressed before firm conclusions can be drawn. First, three of the eight studies focused only on male players [6, 7, 9]. Given the concern over the high incidence of ACL injuries in women, it seems reasonable to suggest that more information is needed regarding playing surface as a potential risk factor in females. Second, there is a need for uniformity in categorizing the severity of injury. Third, injury classification by location and type should be standardized and encouraged. More detailed classification would provide a better understanding of the risk of specific injuries. Fourth, more studies are needed on energy expenditure and fatigue on AT and NG, especially using activities that replicate match play. Given that fatigue can increase the risk of joint injury [24], it is important to understand if player perceptions of increased effort on AT have a physiological basis. Fifth, while preliminary studies suggest that players adjust their style of play on AT, it is not clear if this occurs across levels of play and across genders. Identifying specific movement patterns that either increase or decrease injury risk on AT could impact player training. Finally, more data are needed on young players. As the use of AT in club and school settings increases, a thorough understanding of safety and injury risks in youth and adolescent players is essential.

In this investigation, we found no evidence that playing matches or training on AT raises the risk of soccer players sustaining injury. In fact, the evidence suggests that the risk of some injuries and some subgroups might be lowered. However, until more is known about how issues such as altered playing styles affect injury incidence, it is difficult to make firm conclusions regarding the direct and indirect roles of AT in player safety.

## Figures and Tables

**Table 1:** Descriptions of the studies used in the meta-analyses.

ID	Study	Condition	Gender	Age	Subjects	Study duration	Exposure (hr)	Injuries	Notes
1	Aoki et al. [6]	Both	Male	Youth	332 players	12 months	106,783	484	Ages 12–17 years. Injury location and type reported for training only.

2	Bjørneboe et al. [7]	Both	Male	Adult	14 clubs	4 seasons	261,541	1067	Professional teams in the Norwegian Tippeligaen.

3	Ekstrand et al. [8]	Both	Both	Adult	767 players	Followed teams for 4–38 months	246,475	1492	European elite players. Included “slight” injuries that required players to miss <1 day (~2% of total injuries). Since these could not be extracted, they were included in the present study.

4	Ekstrand et al. [9]	Both	Male	Adult	492 players	Followed teams for 4–32 months	82,874	449	European professional players. Compared two cohorts of players. The cohort of players that trained and played exclusively on grass were omitted from the current study.

5	Fuller et al. [10]	Match	Both	Adult	116 teams 126 teams	1st season 2nd season	79,253	1794	US College players. Utilized the NCAA Injury Surveillance System.

6	Fuller et al. [11]	Training	Both	Adult	116 teams 126 teams	1st season 2nd season	545,842	1592	US College players. Utilized the NCAA Injury Surveillance System.

7	Soligard et al. [12]	Match	Both	Youth	>68,000 players	4 occasions of a single tournament	62,597	2454	Norway Cup youth tournament. Matches played over six consecutive days. Ages 13–19 years. Data for male and female players were pooled.

8	Steffen et al. [13]	Both	Female	Youth	2020 players	8 months	113,023	405	Under-17 age group.

**Table 2:** Adjusted rate ratios for subcategory injuries (95% CI).

	ID	All injuries	Knee injuries	Ankle injuries	Foot injuries^1^	Sprains	Muscle strains
Match	1,2, 3,4, 5,7, 8	0.97 (0.91–1.04)	1.04 (0.91–1.20)	0.98 (0.86–1.12)	0.82 (0.67–1.00)	0.97 (0.87–1.09)	0.85 (0.75–0.97)∗
Training	1,2, 3,4, 6,7, 8	1.04 (0.96–1.13)	0.77 (0.69–0.94)∗	1.07 (0.92–1.24)	1.02 (0.75–1.38)	1.15 (0.99–1.30)	0.90 (0.78–1.03)

Male	1,2, 3,4, 5,6, 7	0.87 (0.81–0.92)∗	0.79 (0.69–1.03)	0.98 (0.87–1.11)	0.92 (0.74–1.15)	1.06 (0.96–1.17)	0.74 (0.66–0.83)
Female	3,5, 6,7, 8	0.85 (0.76–0.94)∗	0.94 (0.77–1.16)	0.83 (0.67–1.02)	1.04 (0.73–1.49)	0.67 (0.56–0.79)∗	0.84 (0.70–1.02)

Youth	1,7, 8	0.90 (0.82–1.00)∗	0.90 (0.66–1.23)	0.89 (0.74–1.08)	0.85 (0.57–1.28)	1.03 (0.84–1.26)	0.54 (0.36–0.81)∗
Adult	2,3, 4,5, 6	0.85 (0.80–0.90)∗	0.82 (0.72–0.92)∗	0.89 (0.79–1.00)	0.96 (0.80–1.16)	0.89 (0.81–0.98)∗	0.79 (0.72–0.87)∗

∗ *P* < 0.05. ^1^Studies 1, 4, and 8 did not define foot injuries.

## References

[1] Cawley P. W., Heidt R. S., Scranton P. E., Losse G. M., Howard M. E. (2003). Physiologic axial load, frictional resistance, and the football shoe-surface interface. *Foot and Ankle International*.

[2] Livesay G. A., Reda D. R., Nauman E. A. (2006). Peak torque and rotational stiffness developed at the shoe-surface interface: the effect of shoe type and playing surface. *The American Journal of Sports Medicine*.

[3] Bowers K. D., Martin R. B. (1975). Cleat surface friction on new and old astroturf. *Medicine and Science in Sports and Exercise*.

[4] Powell J. W., Schootman M. (1992). A multivariate risk analysis of selected playing surfaces in the National Football League: 1980 to 1989. An epidemiologic study of knee injuries. *The American Journal of Sports Medicine*.

[5] Fuller C. W., Ekstrand J., Junge A. (2006). Consensus statement on injury definitions and data collection procedures in studies of football (soccer) injuries. *The British Journal of Sports Medicine*.

[6] Aoki H., Kohno T., Fujiya H. (2010). Incidence of injury among adolescent soccer players: a comparative study of artificial and natural grass turfs. *Clinical Journal of Sport Medicine*.

[7] Bjørneboe J., Bahr R., Andersen T. E. (2010). Risk of injury on third-generation artifi cial turf in Norwegian professional football. *The British Journal of Sports Medicine*.

[8] Ekstrand J., Hägglund M., Fuller C. W. (2011). Comparison of injuries sustained on artificial turf and grass by male and female elite football players. *Scandinavian Journal of Medicine and Science in Sports*.

[9] Ekstrand J., Timpka T., Hägglund M. (2006). Risk of injury in elite football played on artificial turf versus natural grass: a prospective two-cohort study. *The British Journal of Sports Medicine*.

[10] Fuller C. W., Dick R. W., Corlette J., Schmalz R. (2007). Comparison of the incidence, nature and cause of injuries sustained on grass and new generation artificial turf by male and female football players. Part 1: match injuries. *The British Journal of Sports Medicine*.

[11] Fuller C. W., Dick R. W., Corlette J., Schmalz R. (2007). Comparison of the incidence, nature and cause of injuries sustained on grass and new generation artificial turf by male and female football players. Part 2: training injuries. *The British Journal of Sports Medicine*.

[12] Soligard T., Bahr R., Andersen T. E. (2012). Injury risk on artificial turf and grass in youth tournament football. *Scandinavian Journal of Medicine and Science in Sports*.

[13] Steffen K., Andersen T. E., Bahr R. (2007). Risk of injury on artificial turf and natural grass in young female football players. *The British Journal of Sports Medicine*.

[14] Villwock M. R., Meyer E. G., Powell J. W., Fouty A. J., Haut R. C. (2009). Football playing surface and shoe design affect rotational traction. *The American Journal of Sports Medicine*.

[15] Ford K. R., Manson N. A., Evans B. J. (2006). Comparison of in-shoe foot loading patterns on natural grass and synthetic turf. *Journal of Science and Medicine in Sport*.

[16] Potthast W. (2010). Motion differences in goal kicking on natural and artificial soccer turf systems. *Footwear Science*.

[17] McGhie D., Ettema G. (2013). Biomechanical analysis of surface-athlete impacts on third-generation artificial turf. *The American Journal of Sports Medicine*.

[18] Andersson H., Ekblom B., Krustrup P. (2008). Elite football on artificial turf versus natural grass: movement patterns, technical standards, and player impressions. *Journal of Sports Sciences*.

[19] Ronkainen J., Osei-Owusu P., Webster J., Harland A., Roberts J. (2012). Elite player assessment of playing surfaces for football. *Procedia Engineering*.

[20] di Michele R., di Renzo A. M., Ammazzalorso S., Merni F. (2009). Comparison of physiological responses to an incremental running test on treadmill, natural grass, and synthetic turf in young soccer players. *Journal of Strength and Conditioning Research*.

[21] Hughes M. G., Birdsey L., Meyers R. (2013). Effects of playing surface on physiological responses and performance variables in a controlled football simulation. *Journal of Sport Science*.

[22] Nédélec M., McCall A., Carling C., le Gall F., Berthoin S., Dupont G. (2013). Physical performance and subjective ratings after a soccer-specific exercise simulation: comparison of natural grass versus artificial turf. *Journal of Sport Science*.

[23] Sassi A., Stefanescu A., Menaspa P., Bosio A., Riggio M., Rampinini E. (2011). The cost of running on natural grass and artificial turf surfaces. *Journal of Strength and Conditioning Research*.

[24] McLean S. G., Samorezov J. E. (2009). Fatigue-induced acl injury risk stems from a degradation in central control. *Medicine and Science in Sports and Exercise*.

